# Relationship between sociodemographics, loss of income, and mental health among two-spirit, gay, bisexual, and queer men in Manitoba during the COVID-19 pandemic

**DOI:** 10.1371/journal.pone.0278382

**Published:** 2022-12-09

**Authors:** Rusty Souleymanov, Sana Amjad, Samantha Moore, Jared Star, Albert McLeod, Michael Payne, Laurie Ringaert, Linda Larcombe, Gayle Restall

**Affiliations:** 1 Faculty of Social Work, University of Manitoba, Winnipeg, MB, Canada; 2 Manitoba HIV-STBBI Collective Impact Network, Winnipeg, MB, Canada; 3 Department of Community Health Sciences, Rady Faculty of Health Sciences, University of Manitoba, Winnipeg, MB, Canada; 4 Two-Spirit Consultants, Inc., Winnipeg, MB, Canada; 5 Nine Circles Community Health Centre, Winnipeg, MB, Canada; 6 Department of Internal Medicine, Rady Faculty of Health Sciences, University of Manitoba, Winnipeg, MB, Canada; 7 Department of Occupational Therapy, Rady Faculty of Health Sciences, University of Manitoba, Winnipeg, MB, Canada; University of Georgia, UNITED STATES

## Abstract

This study examined the relationship between loss of income due to the COVID-19 pandemic and worsening mental health among a sample of 366 Two-Spirit, gay, bisexual, queer (2SGBQ+) men in Manitoba. Data were drawn from a cross-sectional online survey among 2SGBQ+ men in Manitoba. Logistic regression assessed the relationship between sociodemographics, loss of income due to COVID-19 (independent variable) and worsening of mental health (analytic outcome). Among all respondents in the sample (*N* = 366), 55% indicated worsening of their mental health. In logistic regression, compared to participants who did not experience any loss of income, those who experienced loss of income due to the COVID-19 pandemic were significantly more likely to report worsening mental health (*Adjusted Odds Ratio [AOR]* = 8.32, *95% Confidence Interval[CI]* = 3.54–19.54). Compared to participants who self-identified as gay, bisexual-identifying participants were less likely to report worsening mental health (*AOR* = .35, *95%CI* = 0.13–0.96). Finally, as compared to participants who were married or partnered, participants who were dating (*AOR* = 3.14, *95%CI* = 1.60–6.17), single (*AOR* = 4.08, *95%CI* = 1.75–9.52), and separated/divorced/widowed (*AOR* = 15.08, *95%CI* = 2.22–102.51) were all significantly more likely to report experiencing a worsening of mental health due to the COVID-19 pandemic. This study highlights the need to develop robust public strategies for sub-populations of 2SGBQ+ men (non-gay identified sexual minorities and 2SGBQ+ men who may be more socially isolated). Specific targeted and tailored public health interventions designed with the unique needs of 2SGBQ+ men in Manitoba may be required to increase their access to socio-economic and mental health supports.

## Introduction

The COVID-19 pandemic is reinforcing and exacerbating mental health inequities among vulnerable populations, such as two-spirit, gay, bisexual, queer (2SGBQ+), cisgender and transgender men and other men who have sex with men. In Canada, 2SGBQ+ men are already at a disproportionately high risk for a variety of negative mental health outcomes, such as depression, anxiety, loneliness, and social isolation [1–9].

According to the results of the Manitoba Two-Spirit, Gay, Bisexual, and Queer Men’s Health Study, 52% of participants (*n* = 410) reported that they needed help with problems like anxiety, depression, and suicidal thoughts [10].

Research suggests that mental health stressors may be greater for vulnerable populations, including Two-Spirit, lesbian, gay, bisexual, transgender, queer, and intersex (2SLGBTQIA+) individuals, who report worse mental health outcomes as a result of discrimination, stigma, and experiences of rejection based on their sexual orientation or gender identity [11–13].

Research also suggests that 2SGBQ+ men are 3 times more likely to report depression relative to individuals in the general population with comparable levels of mental health challenges and of similar socio-economic status [14]. Gay men also report experiences of anxiety and mood disorders 3 times more often, and bisexual men roughly 2.5 times more often, than heterosexual men [14]. A Canadian survey (*n* = 2,450) completed between 2017–2019 found that one in four 2SGBQ+ men rated their mental health as poor in the last six months [14]. Moreover, Canadian research conducted in 2018 found that 31% of cisgender gay and bisexual men and 46% of trans-men had thought about taking their life in the previous year [15].

Research conducted in the United States shows that social isolation, limited access to friends and partners and sheltering in hostile (homophobic and transphobic) home environments during COVID-19 is associated with poorer mental health outcomes among 2SGBQ+ men [16–19]. Another US-based study of 1,000 gay and bisexual men found that COVID-19 was having an adverse impact on their mental health, economic security, substance use, quality of life, social isolation, and access to health services [20].

A global study of cisgender sexual minority men showed that men who experienced employment loss due to COVID-19 also reported higher rates of depression and anxiety relative to those without employment loss [21]. Research already showed that gay and bisexual men were worse-off financially, compared to heterosexual counterparts even before the COVID-19 pandemic [22]. One US-based study showed that 30.2% of LGBTQ Americans have reported loss of jobs and 17.9% have reported a reduction in their income due to COVID-19 [23]. Similarly the results of a Canadian study on the impacts of COVID-19 on the LGBTQ2+ communities show that 52% of Canada’s LGBTQ2+ households have faced lay-offs or reduced employment as a result of the COVID-19 pandemic, compared to 39% of overall Canadian households and nearly 60% of LGBTQ2+ respondents said they expect their mental health will be negatively affected in the next 2 months [24].

The impacts of COVID-19 on 2SGBQ+ men, in particular the negative impacts of income loss on 2SGBQ+ men’s mental health, are still unknown. There is a dearth of public health research on 2SGBQ+ men’s communities in Manitoba. The goal of this analysis was to examine the impacts of the COVID-19 pandemic on 2SGBQ+ men’s mental health in Manitoba, including the relationship between socio-demographics and economic impacts of COVID-19 on their mental health. We hypothesize that sociodemographic factors (age, ethnicity, sexual orientation identity, relationship status) and loss of income will be associated with worsening mental health due to the COVID-19 pandemic among 2SGBQ+ men in Manitoba.

## Methods

### Study design

The data used in this paper were collected through a cross-sectional online survey method as part of the quantitative phase of *The COVID-19 & Manitoba Two-Spirit*, *Gay*, *Bisexual*, *and Queer Men’s Health Study*. The study was a 2-part community-based research study designed to examine the impacts of COVID-19 on the health and well-being of 2SGBQ+ men in Manitoba. The study was conducted in collaboration with a community advisory committee (CAC) and the research team. The research team consisted of members connected to health and 2SLGBTQI+ community-based organizations (CBOs). These organizations worked hand-in-hand with our team throughout this research project. The CAC consisted of ten 2SGBQ+ men, representing diverse 2SGBQ+ communities and assisted the research team with the development of the survey, recruitment, and data analysis.

#### Theory-driven approach

This study approaches the worsening of mental health of 2SGBQ+ men in Manitoba during the COVID-19 pandemic as a multi-level challenge, requiring a theory-driven approach, and an understanding of a variety of socio-ecological factors at individual and socio-structural levels [25, 26]. Socio-ecological systems theory provides a framework to examine this population’s healthcare access within the context of individual and socio-structural levels [27, 28]. This project was informed by socio-ecological systems theories, which considers that different factors (age, ethnicity, sexual orientation identity, relationship status, income) may operate at multiple levels (individual, interpersonal and structural) to impact this population’s mental health. A socio-ecological approach recognises the interdependence of people and their environment. Social ecological approaches to mental health outcomes explore individual-level factors, interpersonal relationships, and structural-level factors that include socio-economic contexts. Income and sexual orientation identity are examples of socio-ecological factors associated with health outcomes among 2SLGBTQIA+ people [26, 29]. Our theoretical lens informed our methods, the selection of variables for investigation in this project and the approach to theoretical conceptualisation of mental health in the context of the COVID-19 pandemic.

### Recruitment, sampling, and eligibility

**Fig 1:**
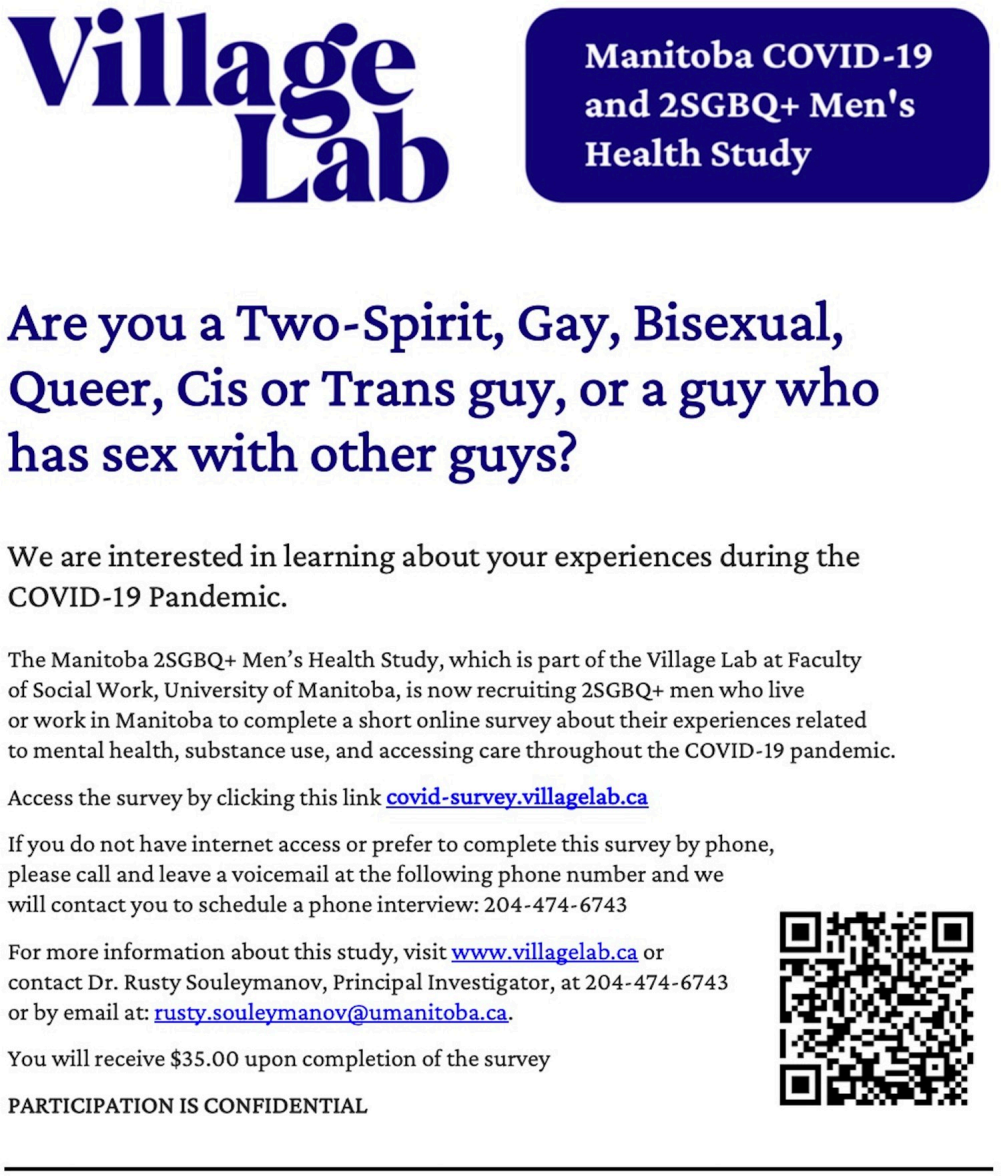
Recruitment flyer.

Participants (n = 366) for the online survey were recruited across Manitoba, using printed flyers (Fig 1) at CBOs, word of mouth, and through Facebook.

CBOs also helped in recruiting 2SGBQ+ men with diverse characteristics and from harder to reach populations, such as Two-Spirit and Indigenous GBQ+ men, racialized 2SGBQ+ men, young adult 2SGBQ+ men, 2SGBQ+ men living in rural settings, and those living with HIV. Participants who did not have access to the internet (six individuals in this study) were given an option to complete this survey by phone. Eligibility included: 1) Identify as a man (cisgender or transgender); 2) Report any sex with another man in the previous 12 months or identify as two-spirit, gay, bisexual, or queer; 3) Be 18 years of age or older; 4) Live in Manitoba. Survey participants were compensated $35 (CAD).

All procedures were approved by the University of Manitoba Research Ethics Board. Informed written consent was obtained from all individual participants included in the study. All data were kept confidential. All procedures performed in studies involving human participants were in accordance with the ethical standard of the institutional and national research committee (University of Manitoba Research Ethics Board 1; Protocol # R1-2021:002 (HS24590) and with the 1964 Helsinki declaration and its later amendments or comparable ethical standards.

### Measures

The online survey included questions on sociodemographics, and impact of COVID-19 on financial wellbeing and mental health. The analytic outcome in this study was worsening of mental health due to COVID-19.

#### Demographics

Sociodemographic measures included: 1) age; 2) race/ethnicity (Black, African, Caribbean; Indigenous; Latin/Latinx, Central American; white/Western European; white/Eastern European); 3) sexual orientation identity (gay, pansexual, bisexual, queer, two-spirit); 4) relationship status (married/partnered, dating, single, separated/divorced/widowed).

#### Loss of income during the COVID-19 pandemic

To understand the experience of any loss of income, we asked participants: “Have you lost any income because of the COVID-19 pandemic?” Answer choices included ‘yes’ and ‘no.’

#### COVID-19 impacts on mental health (outcome variable)

We asked participants if the COVID-19 pandemic has affected their mental health. Individuals who reported that the COVID-19 pandemic has affected their mental health were asked if their mental health was made worse due to the COVID-19 pandemic (with responses ‘yes’ and ‘no’).

### Data analyses

All data analyses were conducted using SPSS 27 (IBM Corp. 2020). First, descriptive analyses and tests were conducted. Second, bivariate analyses were conducted using Chi-Square tests for categorical variables, as well as t-tests for continuous variables. After significant associations were identified in bivariate tests, multivariable analyses were conducted using binary logistic regression with reported adjusted odds ratios (AOR), and 95% confidence intervals (CI). A logistic regression analysis was used to examine the relationship between worsening of mental health due to the COVID-19 pandemic (outcome/dependent variable), sociodemographic (age, ethnicity, sexual orientation identity, relationship status), and loss of income. In the logistic regression model variables were entered using two blocks. In block one, the sociodemographic variables were entered. In block two, the theoretical variable of interest (loss of income) was entered.

## Results

### Participant characteristics and descriptive data

The final sample included 366 2SGBQ+ men. Demographics (participant characteristics) and descriptive data are presented in Table 1. The mean age was 31.3 years (*SD* = 6.0).

**Table 1:** Socio-demographic characteristics and descriptive findings (*N* = 366).

Characteristic	Frequency (n)	Percentage (%)
**Ethnicity**		
White–Western European	185	51.1
Black, African, Caribbean	66	18.2
White–Eastern European	51	14.1
Indigenous	31	8.6
Latino, Latin, Central American	12	3.3
**Sexual Orientation**		
Gay	254	69.4
Pansexual	43	11.7
Bisexual	42	11.5
Queer	8	2.2
Heterosexual	7	1.9
Two-Spirit	7	1.9
**Relationship Status**		
Married or partnered	203	55.5
Dating	91	24.9
Single	62	16.9
Separated, divorced or widowed	10	2.7
**Lost income because of COVID-19 pandemic**		
Yes	300	83.1
No	61	16.9
**Mental health made worse because of COVID-19 pandemic**		
Yes	197	54.4
No	165	45.6

Note. Total numbers vary due to missing data.

### Bivariate analysis

In the bivariate analysis, there was a significant association between worsening of mental health due to COVID-19 pandemic and ethnicity of the participants (*χ*^2^ = 18.07, *df* = 6, *p* = .006), such that White Western European participants were more likely to report worsening mental health. There was also a significant association between the worsening of mental health due to the COVID-19 pandemic and the relationship status of the participants (*χ*^2^ = 10.87, *df* = 3, *p* = .001), with participants who were married or partnered less likely to report worsening mental health. Finally, a significant relationship was found between worsening mental health and loss of income due to the COVID-19 pandemic (*χ*^2^ = 11.71, *df* = 1, *p* = .001).

Furthermore, in the bivariate analysis, there was a significant association between loss of income due to the COVID-19 pandemic and ethnicity of the participants (*χ*^2^ = 16.68, *df* = 6, *p* = .01), where White Western European men were more likely to report loss of income. A significant association was also found between loss of income and relationship status (*χ*^2^ = 20.18, *df* = 3, *p* = .00), so that those who were married or partnered were more likely to experience loss of income.

### Multivariate analyses

#### Factors associated with worsening of mental health due to the COVID-19 pandemic

The results of the logistic regression analyses revealed significant association between relationship status, sexual orientation identity, loss of income and worsening mental health due to the COVID-19 pandemic (see Table 2).

**Table 2:** Multiple logistic regression of socio-demographics and lost income on worsening of mental health due to the COVID-19 pandemic among two-spirit, gay, bisexual, and queer men in Manitoba (*N* = 366).

	B	SE	Sig	AOR	95% CI	Wald statistic
**Socio-Demographic Variables**						
Age	0.00	0.03	0.98	1.00	[0.95, 1.05]	0.01
Race (ref = White/Western European)						
White/Eastern European	-1.11	0.43	0.10	3.80	[0.14, 20.16]	6.61
Black, African, Caribbean	-1.28	0.40	0.79	1.27	[0.13, 7.49]	10.32
Indigenous	-1.18	0.51	0.92	1.10	[0.19, 0.63]	5.34
Latinx, Central American	-1.39	0.85	0.10	0.25	[0.05, 1.32]	2.66
**Sexual orientation (ref = gay/homosexual)**						
Bisexual	-1.04	0.51	0.03	0.33*	[0.11, 0.96]	4.13
Heterosexual	-21.68	16534	1.00	0.01	[0.05, 0.30]	0.01
Queer	-0.03	1.05	0.98	0.97	[0.13, 7.56]	0.01
Pansexual	0.45	0.46	0.32	1.58	[0.64, 3.88]	0.97
Two-Spirit	1.97	1.33	0.14	7.14	[0.52, 97.43]	2.17
**Relationship Status (ref = married/partnered)**						
Dating	1.15	0.34	0.00	**3.28** **	[1.65, 6.51]	11.07
Single	1.41	0.43	0.00	**3.74** **	[1.57, 8.89]	10.59
Separated, divorced or widowed	2.71	0.98	0.01	14.67*	[2.15, 100.04]	7.70
**COVID-19 Variables**						
Lost income due to COVID-19 pandemic	2.12	0.44	0.00	**7.56** **	[3.19, 17.89]	23.68

*Note*. SE = standard error; AOR = adjusted odds ratio; CI = confidence interval; Ref. = reference group

*p* < .05

**p < = .001.

The results of the logistic analysis revealed a significant logistic regression model for worsening of mental health due to the COVID-19 pandemic for this sample of 2SGBQ+ men in Manitoba (*χ^2^* = 75.20, *p* < .00). This model had a very good fit with the sample data *(-2 Log Likelihood* = 352.83, Hosmer and Lemeshow Chi-square test of goodness-of-fit, *χ^2^* = 11.03, *p* > .05, *Nagelkerke R*^*2*^ = 0.28). The model successfully predicted 72.3% of the cases.

With regards to sexual orientation, compared to participants who self-identified as gay, bisexual-identifying participants were less likely to report experiencing a worsening of mental health due to the COVID-19 pandemic (*AOR* = .33, *S*.*E*. = 0.51, *95% CI* = 0.11–0.96, *p* = 0.03). Furthermore, as compared to participants who were married or partnered, participants who were dating (*AOR* = 3.28, *S*.*E*. = 0.34, *95% CI* = 1.65–6.51, *p* < 0.001), single (*AOR* = 374, S.E. = 0.43, 95% CI = 1.57–8.89, *p* < 0.001), and separated/divorced/widowed (*AOR* = 14.67, S.E. = 0.98, 95% CI = 2.15–100.04, *p* = 0.01) were all significantly more likely to report experiencing worsening of mental health due to the COVID-19 pandemic. No other demographics emerged as significant. Finally, compared to participants who did not experience any loss of income, those who experienced loss of income due to the COVID-19 pandemic were significantly more likely to report experiencing worsening of mental health (*AOR* = 7.56, *S*.*E*. = 0.44, *95% CI* = 3.19–17.89, *p* < 0.001).

## Discussion

This study is the first in Manitoba to identify the mental health needs of 2SGBQ+ men during the COVID-19 pandemic. Our findings highlight the severe mental health and economic impacts of COVID-19 experienced by 2SGBQ+ men in Manitoba. Eighty-three percent (83%) of respondents indicated loss of income during this pandemic, and 55% of the sample indicated worsening of their mental health. The findings from this study revealed a significant association between participants’ relationship status, sexual orientation identity, loss of income and worsening of mental health due to the COVID-19 pandemic. Our findings contribute to the body of research that shows the adverse impact of the COVID-19 pandemic on the mental health of sexual and gender minorities [18, 19].

This study adds nuance to this line of research by elucidating differences among different sexual minority identities. Previous research on the mental health impacts of COVID-19 did not differentiate between various sexual minority groups and identities. Research often generalized 2SGBQ+ mens’ mental health outcomes which, in turn, have overlooked the unique mental health needs of bisexual-identifying men [30]. The findings from this study paint a picture where bisexual men were more likely to report worsening mental health during the COVID-19 pandemic compared to participants who self-identified as gay. Although some scholarship suggests that gay and bisexual men do not differ significantly from one another in terms of health practices, other research points out differences between gay and bisexual-identifying men [31, 32]. This finding adds to the knowledge base on the differences between bisexual and gay-identifying men [33]. Bisexual men may be more likely to report worsening mental health during the pandemic for a variety of reasons, including stigma, discrimination, access to services, and biphobia. Previous research showed that bisexual men experience more marginalisation in healthcare and other service contexts compared with gay men [33]. Biphobia and bi-erasure may also discourage some sexual minorities from identifying as bisexual (as opposed to gay) in order to avoid the associated stigma. Given that health services are typically culturally insensitive toward the needs of bisexual men, understanding the mental health disparities (that could have possibly become exacerbated during the COVID-19 pandemic) between gay-identifying and bisexual-identifying men can help inform and develop specific mental health outreach to engage these men in healthcare. Continued public health research efforts are needed to better understand factors that contribute to mental health inequities for bisexual men and to improve mental health outcomes for this population.

Our findings also showed how relationship status may have implications for individuals’ mental health. Being in a relationship (married, or in some form of partnership) has significant implications for how people may have experienced social isolation during this pandemic. The influence of public health restrictions on social and physical connections and social isolation has negative implications for mental health among marginalized groups [34, 35]. Men who are 2SGBQ+ and single, separated, or widowed may experience unfamiliar challenges in receiving informal social support from partners, and may not be able to gather in person or meet other people from their community for social or sexual connections. Other research has already highlighted that COVID-19 restrictions can present a threat to 2SGBQ+ men’s capacity to connect with their communities, including implications for dating and meeting new sexual partners [36]. This, in turn, may precipitate feelings of isolation, reduce their sense of belonging, and result in worsening of their mental health.

Our study found an association between loss of income and mental health. Our findings are consistent with other research which showed that men in a sexual minority who experienced employment loss due to COVID-19 also reported higher rates of depression and anxiety relative to those without employment loss [20]. However, previous research only used loss of employment due to COVID-19 as a proxy for economic impacts of the pandemic, which may not be the most sensitive measure of economic insecurity experienced by individuals (given that individuals may have income that they accrue from sources different than their official job). Our findings contribute to this line of research by showing that loss of income due to COVID-19 is similarly associated with reports of worsened mental health.

Importantly, the study employed a socio-ecological framework to examine the effects of sociodemographics, socio-structural factors (loss of income due to COVID-19 pandemic) and the ways in which these negatively affect the mental health of cisgender and transgender 2SGBQ+ men, emphasizing the multifaceted nature of person-to-environment influences in the socio-ecological framework. This study’s findings therefore contribute to a socio-ecological understanding of worsening mental health among 2SGBQ+ men during the COVID-19 pandemic.

The study results point to several important implications for public health practice, policy, and research. Health professionals who work with 2SGBQ+ men should pay attention to the differences in mental health profiles of bisexual-identifying men and ethno-racialized men in order to better engage these men in public health promotion activities or when designing services for post-pandemic times. Public health intervention efforts may seek to focus on increasing culturally-sensitive and affirming mental health supports, programs, and services for these men.

Our findings also underscore the need to develop more robust strategies for sub-populations of 2SGBQ+ men (non-gay identified sexual minorities, and 2SGBQ+ men who may be more socially isolated, such as single, divorced, or widowed men). Specific targeted and tailored public health interventions designed with the unique needs of these sub-populations may be required to increase their access to economic, social, and mental health supports. Future research needs to continue collecting essential demographic data to allow for meaningful comparative data analysis along lines of diversity and intersectionality, including relationship status, income, and sexual identity. Finally, structural and policy changes that prioritize economic and social equity, public health and those that address systemic barriers that maintain racism, biphobia, and social exclusion affecting 2SGBQ+ men are needed to alleviate the observed disparities.

### Limitations

Limitations of this study include its nonprobability sampling, selection bias, small sample size, and limited generalizability. Our study may be missing 2SGBQ+ men who lack internet access or are not comfortable responding to a 2SGBQ+-focused online survey in their homes. In addition, since we leveraged the networks of CBOs that serve 2SLGBTQIA+ people to facilitate data collection, our findings may best reflect the experiences of 2SGBQ+ men who are affiliated with or use the services of such organizations. Another limitation in our study was not having standardized mental health measures or precise measures for various mental health concerns. Future research needs to examine the impacts of COVID-19 on specific mental health concerns (e.g., anxiety, depression, trauma, suicide) for this population using standardized mental health measures. Finally, while an A priori sample size calculation was not conducted, we relied on a theory-driven data-informed approach, which included an A priori theory-driven analysis plan.

### Conclusions

This study highlighted how COVID-19 may function to deepen existing mental health and socio-economic inequities of 2SGBQ+ men. As the COVID-19 pandemic continues, it is imperative to expand efforts to mitigate the reductions in access to mental health services among 2SGBQ+ men in Manitoba. Continued monitoring of the COVID-19 pandemic’s impacts is of great importance to better understand the evolving economic, social, and mental health needs of this population.
